# Accountability Frameworks in Medical Weight Loss Programs: A Comprehensive Literature Review

**DOI:** 10.7759/cureus.73474

**Published:** 2024-11-11

**Authors:** Olivia C Silveri, Nicholas A Gallardo, Rithi J Chandy, Shenelle A Edwards-Hampton, Steven Feldman

**Affiliations:** 1 Department of Dermatology, Edward Via College of Osteopathic Medicine, Blacksburg, USA; 2 Department of Dermatology, Wake Forest University School of Medicine, Winston-Salem, USA; 3 Department of General Surgery, Atrium Health Wake Forest Baptist, Winston-Salem, USA; 4 Department of Dermatology, Pathology, and Social Sciences &amp; Health Policy, Wake Forest University School of Medicine, Winston-Salem, USA

**Keywords:** accountability, behavioral strategies, patient education, professional education, regimen adherence, social support, weight reduction

## Abstract

Limited adherence to weight loss regimens is a major contributor to the unsuccessful treatment of obesity in patients. Accountability approaches have been used to enhance weight loss program adherence. The purpose of our review is to characterize techniques used to improve patient accountability during weight loss programs. The PubMed database was used to search for studies, analyses, and clinical trials that improved adherence by promoting participant accountability. Articles cited by these references were analyzed, yielding 10 studies. The results were evaluated by comparing efficacy in weight loss, accountability measures used, and the value placed by participants on the program’s focus on accountability measures. Interventions that required social and professional support, planning, physical activity, and an accountability advisor to follow up beyond self-motivation increased the adherence rates of patients. Tools such as online forums and team-based accountability sessions also promoted adherence to long-term weight loss goals. Treatment programs with multiple interventions are optimal when beginning a long-term treatment plan. These accountability strategies may be used in other areas of medicine.

## Introduction and background

Obesity is a global epidemic that affects millions of Americans: nearly one in three US adults (30.7%) are clinically overweight, and more than two in five (42.4%) meet the criteria for obesity [1]. Patients are considered “overweight” or “obese” if they have a body mass index (BMI) above 25 or above 30, respectively [2]. Although patients who are overweight and obese are often able to lose weight temporarily through lifestyle changes, such as dietary modifications and increased physical activity, they often have little long-term success in maintaining weight loss [3]. Common approaches to weight loss begin with counseling from a medical professional, including nutrition and behavioral recommendations to alter the patient’s lifestyle. Education facilitates patient understanding of weight loss through caloric restriction, energy expenditure, and physiological adaptation to diet. Recommended changes often involve eating less, exercising more, and treating comorbid conditions such as smoking, uncontrolled diabetes, lack of sleep, stress, mood symptoms, and disordered eating behavior [4]. Patients are also likely to be offered several resources for losing weight, such as exercise programs, commercial diets, medication interventions, or even surgical interventions for obesity. However, these treatments can be cost- and time-prohibitive for patients [5,6].

Despite the many approaches to weight loss, patients often struggle with adherence [4]. Although dieting can help patients lose weight temporarily, programs focused on adherence through building interpersonal relationships, such as peer-to-peer support under the guidance of a healthcare professional, are more beneficial for total weight loss [4]. In a 2010 study focused on adherence in weight loss trials, attendance to individual/group behavioral sessions and self-monitoring were behavioral indicators of adherence that predicted short- and long-term weight loss [7]. Accountability extends beyond simple adherence, as it refers to the processes by which a person or group understands and takes responsibility for their actions [8]. The purpose of this review is to describe how accountability interventions have been used to promote adherence in weight loss programs focused on interpersonal and social support.

## Review

Methods

 A PubMed search was conducted using the search terms “accountability and weight loss”, “peer-to-peer support for weight loss”, and “weight-loss AND obesity AND online AND social support”. Studies published between January 1, 2011, and January 18, 2024, were included; only patients 18 and older were included. Studies not written in English were excluded. Before applying the inclusion and exclusion criteria, a total of 328 studies were generated with the search terms on the PubMed database. Of the 288 search results generated after the criteria were applied, nine studies were selected for analysis based on their relevance to accountability in weight loss (Table 1). Final articles incorporated at least one of the search terms listed and included a comparison of participants experiencing self-directed weight loss or an interaction with a professional or trained coach throughout their weight loss regimen. Accountability refers to the processes of a person, with additional aid if needed, to take responsibility for their actions [8]. Articles were selected if they involved using the rhetoric of “adherence,” “compliance,” or “accountability,” demonstrating an understanding of a need for weight loss. Studies were chosen based on a primary intention of treating diagnoses of obesity rather than secondary illnesses with a superimposed diagnosis of obesity. These articles included parameters to keep each patient actively involved in their treatment plans, which often involved medical professionals, family members, and the patients themselves. 

**Table 1:** Methods that promote accountability to a weight loss plan from various sources WL: weight loss; iTRE: combining intermittent fasting and early time-restricted eating, defined here as unrestricted eating periods that alternate with water-only fasting periods; SD: standard deviation; WSHS: web-based self-disclosure health support; EHS: email health support

Study	Study type	Intervention type	Outcomes
Bailey-Davis et al. (2022) [9]	Qualitative interview	Researcher interviews with doctors	Many patients with obesity lacked motivation and could benefit from community programs, according to physicians
Reyes et al. (2012) [10]	Qualitative analysis	Researcher interviews with focus group participants	Two years post-weight loss treatment, “regainers” and “maintainers” summarized their successes and failures in their adherence. Regainers regained ≥33% of WL, whereas maintainers regained ≤15% of WL
Liu et al. (2024) [11]	Qualitative study	Six-month weight loss program with two-month follow-up motivational interviews that promoted accountability	Ten out of 15 participants (iTRE n = 4, energy restriction n = 6) lost more than 5% body weight at six months
Carpenter et al. (2022) [12]	Randomized controlled trial	Additional interventionist support	Added support improved consistency with the weight loss plan, but self-monitoring proved to be insufficient. p = 0.006, additional interventionist support increased adherence with participation to the treatment plan and lengthened period of self-monitoring of dietary intake
Budden et al. (2020) [13]	Randomized controlled trial	Weight loss program including social support through an athletic team environment	In a team environment with a coach, participants were motivated and held accountable by peers and also met online through digital support networks. Average WL of 18.48 kg (SD = 12.39)
Evans et al.(2016) [14]	Online survey	Online survey of weight loss bloggers	Blogging duration among weight loss patients led to a higher degree of weight loss during blogging (p = 0.003)
Das and Faxvaag (2014) [15]	Explorative case study	Social media platforms in addition to physical activity	Social media was used to educate patients. No values found
Imanaka et al. (2013) [16]	Open prospective individual randomized controlled trial	EHS participants exclusively received advice from their dietician, while WSHS participants viewed other participants’ progress when accessing the database	Weight loss was significantly greater in the WSHS group than in the EHS group (p = 0.04)
Metzgar et al. (2015) [17]	Qualitative study, comparative trial	Facilitators and barriers were discussed with focus groups in women over the course of 18 weeks	Improving accountability, mindfulness, planning, nutritional aid, and social support allowed for greater treatment adherence. Between the first focus group and week 18 of the primary intervention study, the following changes occurred: 1.3 kg/m^2^ change in BMI (p < 0.05); 3.2 cm change in hip circumference (p < 0.05); 5.4 cm change in waist circumference (p < 0.001)

Results

Accountability and Weight Loss

Patients attempt weight loss through various methods, which may include joining a weight loss program with an individually tailored treatment plan [10]. However, joining a weight loss plan does not guarantee weight loss. In a 2022 qualitative study on weight management care, some challenges patients encountered in initiating a plan included following clinical guidelines from primary care providers or specialists [9]. Consistency of participation in a weight loss plan may be enhanced via medical providers establishing a treatment team familiar with the guidelines based on the patient's unique needs [9].

Medical professionals can assist patients in their weight loss endeavor in person or via telehealth across various services. In a 2012 qualitative study involving 29 participants, intentional weight loss was analyzed over a two-year span with participants separated into groups of “regainers” and “maintainers” [10]. Both cohorts participated in focus groups led by two moderators. The focus groups involved frequent in-person behavioral health counseling sessions, including discussing patient successes and failures and teaching mindfulness skills. Regainers had difficulty independently meeting diet and physical activity goals, while the maintainers described the ease of their experience by using strategies of positive self-talk, self-weighing, and interacting with the focus group. Both cohorts preferred in-person follow-ups over self-monitoring alone with online access to providers to help them continue their habits learned in the initial program [10]. In a 2023 qualitative study, participants were enrolled in a six-month controlled weight loss program with two-month follow-up interviews that promoted self-efficacy and motivational questions on “ownership” of one’s progress [11]. Participants relied on frequent interviews focused on building their accountability in facing challenges, which concurrently bolstered their adherence to their program. Health professionals who aided in early and routine monitoring were able to positively influence patients’ perspective and desire to sustain behavioral changes. The additional aid from physicians to adjust the intervention to each patient’s lifestyle promoted sustained dietary change [11].

Effective weight loss strategies for patients often involve support from their social environment. Not only medical professionals but also teachers, family members, friends, and others can all influence a weight loss treatment plan [12]. A 2022 study demonstrates that patients with additional social support showed increased consistency with participation in their treatment plan and a longer period of adherence to self-monitoring their dietary intake (p = 0.006). Additional social support can promote consistency of engagement in the treatment regimen, help to identify problematic behaviors, and aid the patient in overcoming barriers [12].

Social platforms can be used as an adjunct to professional interventions, as these platforms can provide information and a sense of camaraderie for patients who are attempting to lose weight. For example, the “MAN v FAT football (MVFF) program” from the U.K. provided a community-based regimen that specifically targeted males with a BMI ≥ 27.5 who were attempting to lose weight [13]. This sport-based program appealed to men in a "friendly competition," team-based, gender-sensitized environment, with the support of a coach. Friendly competition has been seen in group dynamics-based programs as a lead factor in behavioral changes [13,14]. The weight loss coach served as a leader who provided support and helped participants achieve their goals through conversation, reflection, and instruction. During this program, the players received incentives to lose weight as a team, promoting group support. By creating a team environment, players are held accountable for their own weight loss plans through peer relationships and a supportive coach. Many members appreciated that the main activity was playing football, taking the focus off of weight loss. In addition, players fostered their support networks off the field through the messaging platform, WhatsApp (Meta Platforms, Inc., Menlo Park, CA, US), to stay connected [13].

Online support groups can serve as a secondary resource to educate patients on weight loss options. The use of online support groups for medical treatment has become more common during the COVID-19 pandemic because of the limited availability of in-person counseling [15]. In a 2015 analysis conducted in Massachusetts, 194 participants who were active bloggers about their weight loss reported that by maintaining their blogs, they were motivated to achieve their weight loss goals [16]. Blogging duration was correlated to increased weight loss (p = 0.003), and participants felt that a sense of community and accountability was the main factor in their success [16]. An online support forum moderated by a professional may help to improve follow-up rates [17]. As the follow-up rates increased, discussions with counselors created a sense of accountability. In a 2013 study conducted in Japan, web-based self-disclosure health support led to higher weight loss than email health support (p = 0.04) [18]. The email health support group received one-to-one advice, while the web-based self-disclosure patients received counseling from registered dieticians and could compare their progress and dietary changes with those of other patients undergoing weight loss treatment. The additional support network created a community for patients to lean on and connect with as they are undergoing a major change in lifestyle [18]. Additionally, in a 2014 case study conducted in Norway, participants who underwent bariatric surgery were interviewed regarding their interaction with an online discussion forum, a secure eHealth portal, over a period of six months. Patients used the platform to interact with peers and providers and to access information and encouragement [17]. The accessible online network helps providers counter inaccuracies and further promote accountability during and after a treatment plan.

Women may experience different facilitators and barriers than men, which can affect their success with their weight loss goals [19]. In a 2014 study involving 23 women across seven focus groups, the significant variables for weight loss were accountability, social support, planning, diet choices, exercise, and self-motivation. Barriers to weight loss for the women included life transitions, changes in health status, environmental pressures, internal factors, and lack of accountability and social support. While some barriers to weight loss plans such as environmental pressures may not be remediable, improving accountability and social support may enhance the effectiveness of weight loss plans [19]. Treatment regimens and accountability strategies yield effective adherence when tailored to the specific patient, emphasizing personalized patient care.

Discussion

Patients use various strategies to assist in weight loss such as self-weighing. Although this is one of the most used strategies across weight loss plans, it is not effective when used alone (-0.5 kg in three months, 95% CI: -1.3 to 0.3) [20]. In a 2015 meta-analysis of different weight loss strategies across various countries, the implementation of multi-component programs in addition to self-weighing led to more effective weight loss (self-weighing alone: -1.7 kg, 95% CI: -2.6 to -0.8; multi-component program with self-weighing: -3.4 kg, 95% CI: -4.2 to -2.6) [20]. These programs included behavioral interventions, self-regulation strategies, and diet and physical activity logging. The presence of each of these accountability components in the weight loss plan led to greater weight loss (-1.3 kg, p = 0.03) [20].

Humans are social beings and often require social interactions, which in turn creates a sense of accountability to effectively begin and maintain weight loss [18]. Without a band of individuals around them, many patients may become lost in their weight loss plans and lose a sense of community. Web-based self-disclosure health support allowed patients to connect with fellow peers undergoing the same treatment rather than one-on-one professional email health support [18]. With increased surveillance and monitoring of patients through interactions with peers and professional support in a web-based setting, patients developed relationships that further supported their goals in adherence. Although online journaling is utilized heavily by women in documenting weight loss progress (n = 194, 92.3% female patients), this may reduce generalizability for male patients [16]. One recommendation is to alter the platform for both men and women so that the application may be more accessible to use, helping to ensure a patient's comfort level in participating with the application in order to be held accountable by their peers.

Transforming the way male treatment is performed with new strategies may improve accountability and adherence for male weight loss patients. Matching a program to its audience is a key tool in increasing the adherence of patients to a treatment regimen [13]. The MVFF program is one example where involving patients who are more sports-minded may increase their adherence [13]. This 2020 study integrated a two-point system of creating a team environment, providing additional access to support through the online application WhatsApp. When searching for new ways to increase accountability in specific populations, it may be beneficial to survey the patient, or group of patients, before initiating a treatment plan. Social media platforms present an accessible tool for patients to begin the process of weight management, but in-person follow-ups enhance accountability beyond the screen [10]. Therefore, programs with multiple components provide patients and caregivers with dynamic decision-making tools from different approaches to ensure patient treatment adherence and prolonged accountability [13].

While many patients can begin a treatment plan for weight loss, successful maintenance of the plan through changes such as exercise and dietary options can be difficult. In the 2012 study comparing maintainers and regainers who had lost 10% or more of their body weight in a two-year span, regainers were less accountable, lost motivation over time, and became complacent in their regimen [10]. As such, one way to improve accountability in patients, with or without caregiver help, may include more frequent follow-ups involving education on therapy and the successes and failures that may come during the maintenance period.

Caregivers often consider how to best treat patients with more facilitators to treatment while avoiding barriers that make weight loss treatment difficult [19]. However, one limitation of including caregivers in the continuity of care is the concept of understanding [21]. It is important for the patient to fully understand each step of treatment in order to be held accountable. Relying on a caregiver may dilute the accountability between the two parties and cause misunderstandings when discussing treatment adherence and dosing. Another limitation may include a patient having multiple caregivers, which could lead to an inconsistency in the advice of caregivers. This may affect education regarding medication responsibilities and adherence [21]. Accountability can be maximized by creating support groups with additional educational sessions, which can subsequently increase treatment adherence [22]. In addition, treatment adherence may be increased through follow-up calls by the provider [19]. By utilizing various strategies in an approach for weight loss patients, it is possible to maximize treatment adherence. Caregiver follow-ups with medical providers strengthen adherence and overall patient accountability of their weight loss program.

Each study reviewed focused on adherence mechanisms, such as clinical techniques and counseling with team surveillance. Accountability was the variable that affected adherence and was the basis for implementing treatment. Success in adherence was determined by participants' accountability to the treatment regimens, which allowed patients to maintain the desired outcomes after each weight loss regimen. The most successful regimens centered around factors of group incentives, community-based support, and digital access programs, which can all be used to monitor progress in a patient’s weight loss plan, as well as foster a sense of accountability through adherence.

A cross-sectional online survey of healthcare providers who used weight loss programs for their patients analyzed the practitioners' perceptions, experiences, and needs in managing patients with obesity [23]. Practitioners expressed the positive effects of motivating patients during obesity programs and how this solidifies a patient’s adherence to their medical regimen; however, there are barriers to accessing multi-disciplinary care, advanced obesity training among physicians, financing for obesity drugs, and comprehensive obesity management guidelines. We identify two potential limitations of this study. Although this study was based on a retrospective review of previous literature, there is novel and ongoing research to consider, such as the developing topic of weight loss medication and its effects. This topic should be explored as providers begin to incorporate medications into patients’ treatment plans and examine how these may affect the accountability of the patient with adhering to their overall weight loss regimen. Also, this study may be limited in scope, and therefore, ongoing efforts should be made to review patient access to resources and provide additional provider education on weight management guidelines with the intent to pursue increased adherence through accountability measures.

## Conclusions

The success of accountability in weight loss treatment plans could impact the way that patients in other fields of medicine are treated. Patients in any specialty may benefit from programs where focused counseling with interpersonal support may result in increased accountability, as accountability can often be an issue of the patient’s lifestyle and time management. Similar positive outcomes like those seen in weight loss trials with increased accountability can lead to better treatment and prognosis of dermatologic diseases such as atopic dermatitis. In addition, treatment plans that were successful in weight loss patients may be modified to treat dermatology patients through a focus on caregiver education.
